# The Role of Thyrotropin-Releasing Hormone Stimulation Test in Management of Hyperthyrotropinemia in Infants

**DOI:** 10.4274/jcrpe.1985

**Published:** 2015-08-31

**Authors:** Ayça Altıncık, Korcan Demir, Gönül Çatlı, Ayhan Abacı, Ece Böber

**Affiliations:** 1 Dokuz Eylül University Faculty of Medicine, Department of Pediatrics, Division of Pediatric Endocrinology, İzmir, Turkey

**Keywords:** hyperthyrotropinemia, hypothyroidism, thyrotropin-releasing hormone

## Abstract

**Objective:**

Hyperthyrotropinemia, which can be either a permanent or a transient state, is an asymptomatic condition and there is a controversy in management and long-term consequences. The aim of this study was to evaluate the results of thyrotropin-releasing hormone (TRH) test in infants with hyperthyrotropinemia.

**Methods:**

Data of the patients who underwent a TRH test for mildly elevated thyroid-stimulating hormone (TSH) levels between 2004 and 2011 in a single academic pediatric endocrinology unit were retrospectively reviewed from the case files.

**Results:**

Twenty infants (13 female, 7 male) with the median (range) age of 33 days (25-50) were enrolled into the study. The median basal TSH was 7.0 mIU/L (4.9-8.9) and free thyroxine level was 1.4 ng/mL (1.2-1.6) at the time of the TRH test. Thyroid ultrasonography was performed to 10 of the cases, and one of them had thyroid hypoplasia. TRH test revealed normal results in four infants, while sixteen infants had exaggerated response suggestive of primary hypothyroidism. The median follow-up period was 3.5 years (2.3-3.7). Therapy was discontinued in seven cases (2 had normal TRH response, 5 had exaggerated response) with the median age of 3.2 years (2.5-4). Of these seven infants, three had an elevated TSH on follow-up and L-thyroxine was restarted. All of the infants, in whom therapy was restarted, had exaggerated response to TRH.

**Conclusion:**

TRH test response could be a useful diagnostic test to evaluate the persistence of the disease during the infantile age period.

## INTRODUCTION

Congenital hypothyroidism (CH) is one of the most common preventable causes of mental retardation. Early detection and treatment of CH prevents neurological deficits. Infants with free thyroxine (fT4) concentration below the norm for age and/or a venous thyroid-stimulating hormone (TSH) concentration persistently >20 mU/L must be treated with L-thyroxine immediately (1). Since the introduction of screening programs for CH, transient or mild disturbances of thyroid function, especially transient hyperthyrotropinemia, have been reported with variable frequencies (2,3,4). If the venous TSH concentration is ≥6 to 20 mU/L beyond 21 days in a healthy infant with a normal fT4 concentration, there is no consensus on the investigation and treatment of these newborns (1).

Nationwide capillary TSH screening is being conducted in Turkey since 2006 and all newborns with TSH values higher than 15 and 5.5 mU/L in the first and second specimens, respectively, are directed to pediatric endocrinologists. Both serum TSH and fT4 measurements are evaluated at recall examination (5). In most cases, diagnosis of hypothyroidism is not difficult to establish given the low T4 with elevated TSH values. However, some of the newborns may have borderline results and have persistent hyperthyrotropinemia with normal or borderline serum T4 levels during infancy (6).

The aims of the present study were i) to evaluate the thyrotropin response to thyrotropin-releasing hormone (TRH) test in infants with mildly elevated TSH and normal-borderline fT4 levels, ii) to evaluate the clinical usefulness of the TRH test in infants, and iii) to determine the natural course of hyperthyrotropinemia.

## METHODS

We retrospectively reviewed the data of infants who were evaluated in our center in the years between 2004 and 2011 and in whom a TRH test was carried out because of mildly elevated TSH values with normal and/or borderline fT4 levels. Exclusion criteria were having multiple pituitary hormone deficiency or central hypothyroidism. Accordingly, infants with low fT4 levels with inappropriately normal or low TSH levels and a TRH test with impaired TSH release, accepted as a TSH increase of less than 5 mI/L (7), were excluded. Incomplete TRH test results because of haemolysed or inadequate blood samples, underlying severe systemic disease, preterm birth (<36 gestation weeks) and clinical follow-up time shorter than a year were also reasons for exclusion. Data including birth weight, gestation week, family history, age at the time of the TRH test, laboratory and radiological findings during follow-up were recorded from the case files.

A TRH stimulation test was performed after confirming that at least two TSH levels were between 5-20 mIU/L. A baseline blood sample was collected before TRH injection for fT4, TSH, and prolactin (PRL) measurement. TRH (Ferring Pharmaceuticals Ltd, Kiel, Germany; 7 μcg/kg, maximum 200 mg) was administered as an intravenous bolus. Plasma TSH and PRL were measured 15, 45 and 60 minutes after administration of TRH. A peak TSH value higher than 35 mIU/L after TRH stimulation was accepted as an exaggerated response suggestive of primary hypothyroidism and those below 35 mIU/L were considered normal (8).

All hormone assays were performed in the endocrine laboratory of the Dokuz Eylül University Faculty of Medicine. TSH and PRL were measured by immunochemiluminometric assay (Immulite 2500 immunoassay, Siemens Diagnostic Products Corporation). Free T4 was measured by RIA.

### Statistical Analysis

SPSS 13.0 (SPSS Inc., Chicago; IL) was used. Mann-Whitney U-test (non-parametric data) was used for comparisons of numerical variables. Chi-square or Fisher’s exact test were used to compare categorical variables. For all analyses, a two-tailed p<0.05 was considered statistically significant. Results were expressed as n (%) or median (25th-75th percentile).

## RESULTS

**Figure 1:**
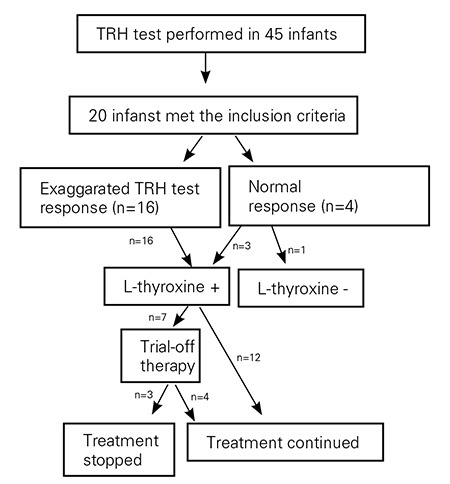
Scheme of the study group

Of the 45 patients who underwent a TRH test, 20 infants (13 female, 7 male) fulfilled the inclusion criteria (Figure 1). There was no history of maternal ingestion of thyroid hormones or goitrogens. The parents of two infants were consanguineous. In four cases, family history revealed hypothyroidism in grandmothers and aunts. Seven infants had nonsensical clinical signs (prolonged jaundice, decreased weight gain, or constipation).

Median age of the infants at the time of TRH testing was 33 days (25-50 days). Four infants had a normal (group 1) and sixteen infants had an exaggerated response to TRH (group 2) (Figure 1). Clinical and laboratory data of the groups are given in Tables 1 and 2.

Bone age was determined in seven cases. One infant with exaggerated TRH test had delayed bone age (absence of distal femoral epiphyses), others were normal. Thyroid volume and morphology were examined by ultrasound in 10 cases, and one of the children had thyroid hypoplasia (thyroid volume was 0.15 mL=-2.89 SDS according to reference values (9).

All patients except one with normal TRH test response received L-thyroxine treatment (Figure 1). The median follow-up period was 3.5 years (2.3-3.7 years). The median age of infants at the last clinic visit was 3.8 years (3.3-4.5 years).

Treatment was discontinued in seven cases (two had a normal TRH response, five an exaggerated response) at a median (range) age of 3.2 years (2.5-4 years) (Figure 1). The L-thyroxine dose was 0.9 mg/kg/d (0.9-1.5 mg/kg/d), median TSH level 2.2 mIU/L (2.1-4.2 mIU/L) and median fT4 level was 1.3 ng/mL (1.1-2.2) at the time of discontinuation. Of these 7 infants, 3 had an elevated TSH on follow-up and L-thyroxine was restarted (Figure 1). All of the infants in whom treatment was restarted had shown an exaggerated response to TRH. In the remaining 4 infants in whom treatment was discontinued, median follow-up period after discontinuation was 1.8 years (0.4-3.8), and the median TSH and fT4 levels at the last clinic visit were 2.1 mIU/L (1.6-2.7) and 1.4 ng/mL (1.1-3.9), respectively. Comparison of the clinical and laboratory data of the infants are given in Table 3.

In twelve infants (one with normal, eleven with exaggerated TRH response), discontinuation had never been tried (Figure 1). The median follow-up period was 3.8 years (2.8-3.9), and the median L-thyroxine dose was 1.9 mg/kg/d (0.9-3.0 mg/kg/d). Of these 12 infants, one had thyroid dysgenesis, eight had occasions of high TSH levels while under L-thyroxine therapy, and three were younger than 3 years old.

## DISCUSSION

Rapaport et al (8) performed a TRH test to 68 infants with subclinical hypothyroidism (SH). Peak TSH values of 35 mIU/L or less were considered as normal, and peak TSH values above 35 mIU/L were considered as hyperresponsive. L-thyroxine replacement was given to all hyperresponsive infants. During long-term follow-up, all infants with normal TRH test results have remained euthyroid. Thirteen of 40 hyperresponsive infants had evidence of thyroid dysfunction in follow-up (elevation of TSH during or after discontinuing treatment) and treatment had to be continued or re-started. The authors have concluded that the TSH response to TRH is a useful tool for the evaluation of infants with mildly abnormal TSH levels and that infants with TSH levels >35 IU/mL after TRH are at risk of hypothyroidism. In our study, we were able to discontinue treatment in only 2 of the 16 infants with exaggerated responses. Treatment was discontinued in 2 of the 4 infants with normal TRH test results. Calaciura et al (10) evaluated thyroid functions in 56 newborns with false positive screening results (blood spot TSH>20 mU/L, normal serum fT4 with normal or borderline high/normal TSH at recall). Infants with normal TSH levels at recall were considered as group 1, and those with mildly elevated TSH levels (5-11.7 mU/L) as group 2. TRH test was carried out in 48 of 56 children. 33% of group 1 and 59% of group 2 infants had exaggerated responses. L-T4 treatment was started in infants in group 2 and was stopped 2-3 months before the initiation of the study, at a time when the infants had reached ages 2-3 years. At 2-3 years old, serum TSH levels were found to be higher than the 99.7th percentile of healthy controls in 50% of the study group. The authors concluded that infants with elevated TSH levels at neonatal screening are at risk of the development of SH in early childhood. However, this study did not report any information on the differences in the outcome of cases with normal or exaggerated responses to the TRH test. In our study, treatment could be discontinued in only two of the 16 infants with exaggerated TRH test results. Both of these infants were younger than 3 years and discontinuation had never been tried. In the remaining 12 infants, therapy could not be discontinued or had to be restarted.

A longitudinal evaluation of 44 of the 56 infants in Calaciura et al’s study was reported later (10,11). Twenty-eight of these 44 (63.6%) children had SH at early childhood (2-3 years old) and in 19 of these 28 (67.8%) children, serum TSH levels persisted elevated at the evaluation between 4-6 years old. When thyroid functions were evaluated at a pre-pubertal age (a mean age of 8 years), high TSH levels were found to persist in 14 of 19 children (50% of the children with SH at early age). Morphological abnormalities of the thyroid gland were frequent in these children. None of the children developed overt hypothyroidism in this study. However, mild hypothyroidism at prepubertal ages was observed in 22% of the children whose recall TSH was less than 5.0 mU/L in the newborn period and in 43% of children whose recall TSH was in the range of 5.0-12.0 mU/L.

In our study, one infant did not receive treatment due to normal TSH and fT4 levels at the TRH test. Treatment could be discontinued in 4 of the 20 infants and TSH levels were normal during the follow-up period of 2.0±1.7 years. In conclusion, in five of the 20 infants with high TSH levels at recall examination, TSH levels were normalized.

SH has been associated with heart diseases and dyslipidemia in adults (12,13). Progression to overt hypothyroidism is reported to be higher in adolescents (14). Very few data exist regarding the long-term outcome in newborns and infants with idiopathic SH without any underlying disease. In these reports, normalization of TSH varied between 39 and 73.6% and 25-58.7% of the patients remained SH during an observation period of 2-5 years. An increased TSH levels (>10 mIU/L) with normal fT4 were determined in 2-24.9% of the patients. Overt hypothyroidism developed in 0.03-13.5% of the children. Possible predictive factors for the development of hypothyroidism were initial TSH level >7.5 mIU/L and female gender (15,16,17). In our study, the clinical and laboratory data of the patients in whom treatment could be discontinued were not significantly different from those of the patients who had to continue treatment. Hence, there were no possible predictive factors to decide the discontinuation of treatment.

Daliva et al (18) followed-up fourteen infants with SH who were recalled from screening test. Six infants underwent TRH testing and had an exaggerated response. All infants were treated with L-thyroxine, and treatment was discontinued in all except one when the infants were 3 years old. Ten children had abnormal thyroid function tests on retesting off therapy and L-thyroxine was restarted. Three children had normal retest thyroid function and underwent a TRH test. Of these, 2 children had exaggerated response and L-thyroxine was restarted. TSH levels were normalized in only one child. All of the children with an exaggerated TRH response had to restart therapy due to persistently elevated TSH levels. In our study, L-thyroxine was restarted to three (two boys, one girl) of the 7 infants in whom the treatment was discontinued; however, overt hypothyroidism did not develop in these infants during the follow-up period without medication.

The major concern in the infants with SH is whether this situation has an effect on the infant’s growth and mental development or not. In studies which have evaluated the natural course of idiopathic SH in children who received no treatment, no effects in height growth and BMI were found throughout the observation period (16). Also, no differences in height and BMI between treated and untreated children with SH were reported (19). We compared the auxological data of the infants with normal and exaggerated TRH test response at the last clinic visit and found no significant differences between the groups. However, it has to be considered that all of the infants in our study had received treatment.

There is limited data regarding the effect of thyroxine therapy on neurocognitive function in children with SH. It is reported that these children have difficulties in focusing their attention and controlling their instinctive responses. Some studies showed that replacement therapy did not have an impact on neuropsychological function. However, duration of L-thyroxine treatment and number of subjects in these studies were very limited (20,21). We routinely do not perform neurodevelopmental or intelligence tests in infants with SH in our department. None of the parents had reported any complaints such as attention disorder in our study group.

Environmental factors such as iodine deficiency, maternal autoimmune factors, and genetic factors like thyroperoxidase (TPO) and TSH receptor (TSH-R) gene mutations can cause SH. Our study design was retrospective and because genetic tests were not performed routinely in these patients, genetic data were absent. Also, although Turkey is an iodine-deficient area, urinary iodine measurements were not performed in our study subjects. These were the limitations of our study.

In conclusion, L-thyroxine treatment could be discontinued in only 2 of the 16 infants with exaggerated TRH test response in this study. Treatment was not restarted in two of the 4 infants with normal TRH test results. TRH response to TRH stimulation may be a useful diagnostic test for the evaluation of these infants.

## Figures and Tables

**Table 1:**
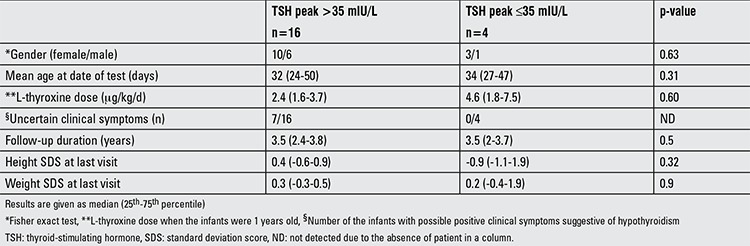
Clinical, laboratory, and auxological parameters of the infants in groups 1 (normal thyrotropin-releasing hormone test result) and 2 (exaggerated thyrotropin-releasing hormone test result)

**Table 2:**
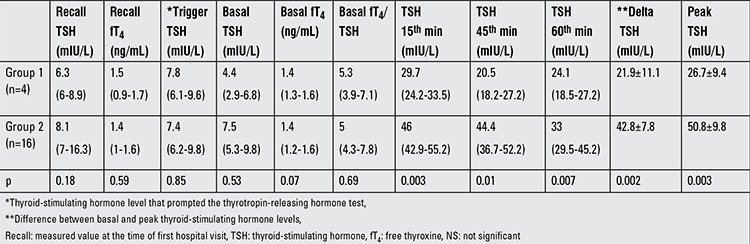
Comparison of thyrotropin-releasing hormone test parameters of the infants in groups 1 and 2

**Table 3:**
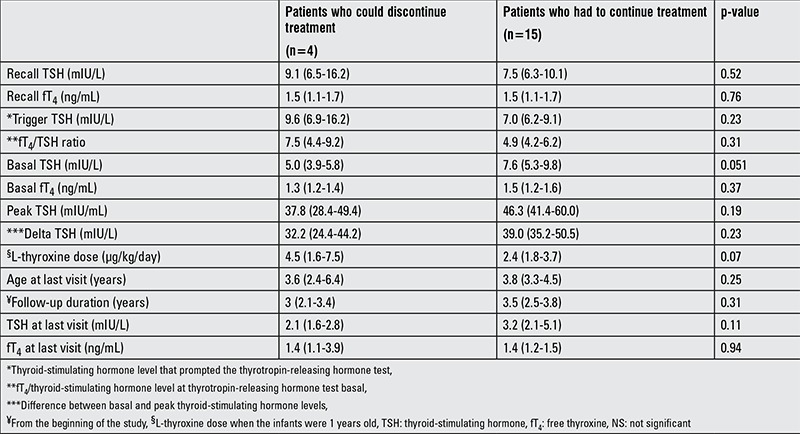
Clinical and laboratory data of the patients who could discontinue and those who could not discontinue treatment
